# Undergraduate nursing students’ knowledge, attitudes and self‐efficacy regarding palliative care in China: A descriptive correlational study

**DOI:** 10.1002/nop2.635

**Published:** 2020-10-13

**Authors:** Yinghua Zhou, Qiao Li, Wei Zhang

**Affiliations:** ^1^ Faculty of Nursing School of Medicine Jiangsu University Zhenjiang China; ^2^ Department of Sociology Jiangnan University Wuxi China

**Keywords:** attitude to death, knowledge, nursing, palliative care, self‐efficacy, students

## Abstract

**Aim:**

To describe nursing students’ knowledge, attitudes and self‐efficacy about palliative care and to examine the associations between these variables in China.

**Design:**

A descriptive correlational study.

**Methods:**

Undergraduate nursing students (*N* = 187) at the end of third year of education from a university were surveyed. Measurements included the Chinese versions of the Palliative Care Quiz for Nursing, the Death Attitude Profile‐Revised, the Frommelt Attitude Toward Care of the Dying Scale and the Palliative Care Self‐Efficacy Scale. Descriptive and correlational analyses were performed.

**Results:**

Although most nursing students had favourable attitudes towards death and caring for the dying, students had low level of knowledge and self‐efficacy regarding palliative care, suggesting the need for integrating palliative care education into nursing curriculum in China. Moreover, special attention should be paid to psychosocial and spiritual care teaching and preparing students to psychologically deal with the challenges in the process of patient's dying.

## 1 INTRODUCTION

“Palliative care is an approach that improves the quality of life of patients and their families facing the problem associated with life‐threatening illness, through the prevention and relief of suffering by means of early identification and impeccable assessment and treatment of pain and other problems, physical, psychosocial and spiritual” (World Health Organization, 2006). With an increase in the ageing population in China, more elderly people are living with incurable and life‐limiting diseases (Hu & Feng, 2016). In addition, more patients are requiring the quality palliative care due to the improved cancer survival rate (Park et al., 2019). However, palliative care is still limited in China and the shortage of trained healthcare providers is a vital barrier that hinders the development of palliative care (Yin et al., 2017). In China, there are very few contents related to palliative care in nursing textbooks and palliative care training is generally inadequate for nursing students (Li et al., 2015). Therefore, it is needed to identify the status of palliative care education in nursing students before designing palliative care course in nursing schools.

## 2 BACKGROUND

Nursing students, who will be future nurses, should be well educated to deliver quality palliative care. In general, lack of knowledge is a vital obstacle in providing palliative care (Al Qadire, 2014). Many studies investigating the palliative care knowledge of undergraduate nursing students revealed that knowledge is still inadequate (Aboshaiqah, 2020; Al Qadire, 2014; Dimoula et al., 2019; Jiang et al., 2019). For example, Aboshaiqah (2020) found that nursing students in Saudi Arabia had low level of knowledge regarding palliative care.

In addition, nurses’ attitudes towards caring for the dying also influence the quality of palliative care (Cevik & Kav, 2013). There were some studies investigating nursing students’ attitudes towards caring for the dying, but the results were varied from unfavourable to favourable across different countries (Abu‐EL‐Noor & Abu‐EL‐Noor, 2016; Arslan et al., 2014; Grubb & Arthur, 2016; Henoch et al., 2017). These varied results might be due to that many factors influence the participants’ attitudes towards caring for dying patients such as level of education, education in palliative care, previous experience in caring for dying patients, attitudes towards death, cultural contexts and religious belief (Arslan et al., 2014; Braun et al., 2010; Grubb & Arthur, 2016; Iranmanesh et al., 2008; Wang et al., 2018).

Moreover, nurses’ attitudes towards death might influence their attitudes towards caring for dying patients (Braun et al., 2010; Iranmanesh et al., 2008) and can have an impact on the quality of palliative care which they provide to the patients. However, previous studies showed conflicting evidences about the association between nurses’ attitudes towards death and attitudes towards caring for the dying (Braun et al., 2010; Iranmanesh et al., 2008; Wang et al., 2018; Wessel & Rutledge, 2005). For example, Wessel and Rutledge (2005) found that death attitude (death avoidance) was significantly negatively correlated with attitudes towards caring for the dying in southern California nurses. However, Iranmanesh et al. (2008) and Wang et al. (2018) did not find significant relationship between death avoidance and attitudes towards caring for the dying in Iranian nurses and in Chinese nurses.

Self‐efficacy is also thought to be a critical determinant of palliative nursing (Desbiens et al., 2012). Self‐efficacy refers to the confidence that the individuals have in their ability to perform a specific behaviour or skill (Bandura, 1997). Based on Bandura's social cognitive theory, if a nursing student believes that he or she has the ability to provide palliative care and that the outcome of this palliative care would improve patient's quality of life, he or she will be more likely to deliver palliative care to the patient. Higher self‐efficacy also improves decision‐making and information sharing (Bandura, 1997). However, a study revealed that graduating nursing students in bachelor degree programme were not adequately educated to achieve the required capabilities in palliative care and further education is needed to improve students’ self‐efficacy regarding palliative care (Henderson et al., 2016).

In sum, nursing students’ palliative care knowledge, attitudes towards death and caring for the dying and self‐efficacy are indicators for the quality of palliative care implementation. A baseline assessment of these variables is valuable for knowing the status of palliative care education and further developing palliative care course. Remarkably, Zhou and Zhang (2015) investigated nursing students’ knowledge, attitudes and self‐efficacy regarding pain management. They found that nursing students had low level of knowledge and attitudes regarding pain management but reported moderate level of self‐efficacy. This revealed a very dangerous situation: most student nurses thought that they were able to manage pain even though they had limited knowledge about pain management. The incorrect self‐evaluation may make students do not want to get further education about pain management and hamper effective pain management. In addition, Pfister et al. (2013) measured 130 nursing home professionals’ knowledge and self‐efficacy related to palliative care in Germany and found low level of knowledge and self‐efficacy. However, self‐efficacy was negatively correlated with age and working experience, which might imply that the difficulty of palliative care skills was underestimated by inexperienced care workers. Moreover, insufficient knowledge may make nursing students feel unprepared and stressful regarding palliative care providing, which perhaps leads to the development of negative attitudes towards caring for the dying (Mutto et al., 2010). Therefore, a deep understanding of nursing students’ knowledge, attitudes and self‐efficacy regarding palliative care is needed. However, previous studies mainly focused on nursing students’ or nurses’ palliative care knowledge and attitudes (Dimoula et al., 2019; Kassa et al., 2014; Kim & Hwang, 2014) or palliative care knowledge and self‐efficacy (Brazil et al., 2012; Pfister et al., 2013). One study investigated oncology nurses’ palliative care knowledge, attitudes towards caring for the dying and perceived self‐competence in Vietnam (Nguyen et al., 2014), yet attitudes towards death were not included in this study.

In China, although there are some studies investigating nursing students’ palliative care knowledge (Jiang et al., 2019; Li et al., 2015) or attitudes towards death and caring for the dying (Liu et al., 2015; Peng et al., 2018), students’ self‐efficacy is not investigated and the associations among these variables are also not explored. Thus, the aim of this study is to assess nursing students’ knowledge, attitudes and self‐efficacy about palliative care and explore the associations between these variables.

### 2.1 Context

Cain et al. (2018) illustrate the strong influence of culture on palliative care in four aspects: meanings of suffering, preferences for care, decision‐making processes and communication patterns. Although death and dying are a universal fear, peoples’ attitudes towards death might be different across diverse cultures. Chinese traditional culture is deeply influenced by Confucianism, which emphasizes the value of “life” and ignores the discussion of “death.” Therefore, talking about death is taboo and most Chinese people avoid discussing about death openly (Hsu et al., 2009). Meanwhile, under the family oriented culture in China, family commonly plays a more important role in decision‐making process than patient (Zheng et al., 2015). When a patient is diagnosed with terminal disease, the medical staff should first inform the family members of patient's condition and the family members will decide whether to inform the patient (Sun et al., 2011). Consequently, due to family's willing to maintain patient's hope and the death taboo culture, many cancer patients had never been told about their diagnosis even at the terminal stage (Zheng et al., 2015). The death taboo culture also makes it difficult for nurses to communicate effectively with dying patients and their families (Zheng et al., 2015).

In China, the initial nursing education comprises three levels including the 3‐year secondary diploma programmes, the 3‐year advanced diploma programmes and the 4‐year or 5‐year baccalaureate programmes (You et al., 2015). There were 982 secondary diploma programmes, 339 advanced diploma programmes and 216 baccalaureate programmes in China in 2012 (You et al., 2015). However, with the development of economic and social society, the number of baccalaureate programmes is gradually increasing. By the end of 2018, the total number of registered nurses in China has exceeded 4 million. Initially, nursing education adopted the bio‐medical model in China. Currently, the nursing curriculum framework changed from medical focus to nursing process‐oriented model (Wang et al., 2016). However, study reported that nursing education in universities is less related to palliative care knowledge and nursing students’ palliative knowledge is generally not improved in the process of clinical practice (Li et al., 2015).

Research questions:

1. What is the level of nursing students’ palliative care knowledge and self‐efficacy?
2. What are the nursing students’ attitudes towards death and attitudes toward caring for dying patients?
3. Are there any relationships between the palliative care knowledge, self‐efficacy, attitudes towards death and attitudes towards caring for dying patients?

## 3 THE STUDY

### 3.1 Design

This was a descriptive, cross‐sectional and correlational study.

### 3.2 Setting and participants

A convenience sampling method was used. A total of 198 undergraduate nursing students at the end of third year of education in faculty of nursing, Jiangsu University were chosen. The students had completed all nursing coursework and already had some practical experience with different patient groups in hospital. Jiangsu University is a comprehensive university in Jiangsu province, and it is located in the east region of China, which is more developed than the west and middle regions. The 4‐year bachelor nursing curriculum in Jiangsu University, as most other nursing colleges in China, consists of three years of coursework on basic and clinical nursing sciences and one year of clinical practice. There is no elective course or mandatory education in palliative care in the nursing curriculum. Only few contents related to end‐of‐life care are arranged in the form of a chapter in other courses, such as Nursing foundation, Nursing ethics, Community nursing, and the total number of class hours is limited to about 5. The nursing curriculum framework is nursing process‐oriented model, and the current teaching method is mainly classroom teaching. Recently, teachers have been required to use various teaching methods such as problem‐based learning, case‐based learning, simulation teaching and so on. However, it may take a certain amount of time for the pedagogical change in the university.

### 3.3 Instruments

The questionnaires used in this study included five parts: (a) demographic questionnaire; (b) the Chinese version of the Palliative Care Quiz for Nursing (PCQN‐C); (c) the Chinese version of the Death Attitude Profile‐Revised (DAP‐R‐C); (d) the Chinese version of the Frommelt Attitude Toward Care of Dying Patients Scale (FATCOD‐C); and (e) the Chinese version of the Palliative Care Self‐Efficacy Scale. The Chinese versions of the questionnaires were used with the permission of its author.

The PCQN, designed by Ross et al. (1996), is a widely used and well‐validated tool for assessing palliative care knowledge. The PCON was translated into Chinese and validated in nurses by Zou et al. (2006). Compared with the original PCQN, there was no major change in the PCQN‐C. The PCQN‐C has 20 items categorized into three subscales including the philosophy of palliative care (4 items), management of pain and symptoms (13 items) and psychosocial and spiritual care (3 items). The respondents select “true,” “false” or “do not know” for each item. We assigned a 1 score for correct answer and a 0 score for wrong and “don't know” answer. Total scores for the PCQN‐C range from 0–20. In this study, the internal consistency reliability of the PCQN‐C measured by KR‐20 was 0.60.

The DAP‐R, developed by Wong et al. (1994), is widely used for assessing respondents’ attitudes towards death. The DAP‐R, totally 32 items, is composed of five subscales. Fear of death measures the respondent's negative thoughts and feelings about death (7 items: 1, 2, 7, 18, 20, 21 & 32). Death avoidance measures the respondent's attempts to avoid thoughts about death (5 items: 3, 10, 12, 19 & 26). Neutral acceptance indicates that the respondent views death as a natural part of life and neither welcomes nor fears death (5 items: 6, 14, 17, 24 & 30). Approach acceptance describes that the respondent views death as a pathway to a better afterlife (10 items: 4, 8, 13, 15, 16, 22, 25, 27, 28 & 31). Lastly, escape acceptance indicates that the respondent considers death as an escape from a painful and suffering life (5 items: 5, 9, 11, 23 & 29). Tang et al. (2014) translated the DAP‐R into Chinese, and the validity and reliability were proved among 126 nurses. The only difference between the original DAP‐R and the DAP‐R‐C is that the original DAP‐R used a 7‐point Likert Scale, but the DAP‐R‐C used a 5‐point Likert Scale from 1 (strongly disagree) to 5 (strongly agree). The score for each subscale is the mean score of its items; scores range from 1–5. A higher score for one subscale indicates that the respondents show a stronger tendency in this subscale. In this study, subscale reliability of the DAP‐R‐C was tested with Cronbach's alpha coefficient varying from a low of .62 in neutral acceptance to a high of .83 in death avoidance.

The FATCOD (Frommelt, 1991) is commonly used to evaluate respondents’ attitudes towards caring for dying patients and their families. The FATCOD was translated into Chinese and validated by Tang et al. (2015), and no major changes were made. The FATCOD‐C consists of 30 items with 15 positively worded items and 15 negatively worded items. The FATCOD‐C is a 5‐point Likert scale. Positively worded items are scored from 1 (strongly disagree) to 5 (strongly agree). Scores for negatively worded items are reversed. Therefore, total possible scores for the FATCOD‐C range from 30–150; higher scores reflect more positive attitudes. An overall score was also computed and converted to percentage. Student who has a score of higher than 65% of the total possible suggests that he/she has a positive attitude towards caring of the dying; student who has a score of lower than 50% of the total possible suggests that he/she has a negative attitude (Grubb & Arthur, 2016). In this study, Cronbach's alpha coefficient of the FATCOD‐C was .72.

Palliative Care Self‐Efficacy Scale (Phillips et al., 2011) was developed and validated in nurses. This scale measures respondents’ perceived capabilities to provide palliative care, and it has two subscales including psychosocial support (6 items, items 1–6) and symptom management (6 items, items 7–12). This scale is a 4‐point grading scale with 1 representing “need further basic instruction,” 2 representing “confident to complete with close supervision,” 3 representing “confident to complete with minimal consultation” and 4 representing “confident to complete independently.” The respondents rate their confidence in their ability to complete each palliative care task. The Palliative Care Self‐Efficacy Scale was translated into Chinese using the standard forward–backward procedure in this study. Then, four nursing experts from Geriatrics Department and Oncology Department in hospital reviewed the content of the Chinese version of this scale. The content validity index was found to be 1.0. Finally, no major changes were made. Pilot testing of the Palliative Care Self‐Efficacy Scale was conducted on 25 fourth year undergraduate nursing students who practised in the affiliated hospital of Jiangsu University in April 2018. Cronbach's alpha of the Chinese version of the Palliative Care Self‐Efficacy Scale and its subscales ranged from 0.58–0.78 from the pilot testing and from 0.67–0.82 from the whole study testing. The mean total self‐efficacy score was computed, and possible scores ranged from 1–4.

### 3.4 Data collection

In June of 2018 and 2019, a total of 198 undergraduate nursing students who were at the end of third year of education in faculty of nursing, Jiangsu University, were approached. The first researcher who was a teacher of the participants approached the students at the end of one of their classes to determine their willingness in participation. The purpose of the study and instructions to fill the questionnaires were explained to the students. Students completed the questionnaires independently, and no reference materials were available. The questionnaires were administered to the students who agreed to participate and returned to the first researcher when completed. Completion of the questionnaires took 20–25 min, and no compensation was given to the students. The whole process took about 45 min.

### 3.5 Data analysis

The SPSS (version 20.00) was used for analysis. Descriptive statistics were used for summarizing demographic characteristics and the study variables. Pearson's correlation analysis was performed to examine the relationships between the study variables. *P*‐value <.05 was set at for significance. The correlation magnitude was based on the Munro's classification (little if any [0.00–0.25], low [0.26–0.49], moderate [0.50–0.69], high [0.70–0.89] and very high [0.90–1.00]) (Munro, 2001).

### 3.6 Ethics

Ethics approval was obtained from the Medical School Ethics Committee at Jiangsu University. Since the first researcher also was the one who graded the students on one course, this way of collecting data had potential risk related to authority and power which might put a pressure on the students to participate. To reduce the potential risk, the researcher explained to the students that the participation in this study was voluntary and students did not need to worry about the negative consequences of non‐participation or withdrawal (e.g. students’ class performance would not be affected by whether they participated or not.). Signed informed consent was obtained. There was no identifiable personal information in the questionnaires. All the information in the questionnaires was only used for the purpose of this study. Thus, the confidentiality and anonymity of information provided were guaranteed.

## 4 RESULTS

### 4.1 Participants

Of the 198 students invited, four students chose not to participate, and seven students were excluded from the analysis because of incomplete questionnaires. Finally, the sample consisted of 187 students (response rate 94.4%), including 5.3% males and 94.7% females. The mean age of students was 21.34 (*SD* 0.70). 92% of students were not religious. 4.8% of students experienced severe illness before and 11.8% experienced death of a significant other. 47.1% of students were willing to care for the dying patients and 77% wanted to get more palliative care education.

### 4.2 Palliative care knowledge

The mean total knowledge score was 9.04 (*SD* 2.33) on the PCQN‐C ranging from 2–16 and the average correct rate of knowledge was 45.2%, indicating a lack of knowledge regarding palliative care among nursing students. In the PCQN‐C, the category which scored the highest percentage of correct answers was management of pain and symptoms (50.76%), and the category which scored the lowest was the psychosocial and spiritual care (25.67%) (Table 1). Item 4 “Adjuvant therapies are important in managing pain” got the most correct answers and item 5 “It is crucial for family members to remain at the bedside until death occurs” got the fewest correct answers.

**TABLE 1:** The results of the Chinese version of the Palliative Care Quiz for Nursing

Scale item	Subscale	Correct *N* (%)	Incorrect *N* (%)	Don't know *N* (%)	Correct rate
	Philosophy and principle of palliative care				41.71%
1	Palliative care is only appropriate in situations where there is evidence of a downhill trajectory or deterioration (F)	124 (66.3)	44 (23.5)	19 (10.2)	
9	The provision of palliative care requires emotional detachment (F)	106 (56.7)	43 (23.0)	38 (20.3)	
12	The philosophy of palliative care is compatible with that of aggressive treatment (T)	66 (35.3)	80 (42.8)	41 (21.9)	
17	The accumulation of losses renders burnout inevitable for those who seek work in palliative care (F)	16 (8.6)	150 (80.2)	21 (11.2)	
	Management of pain and symptoms				50.76%
2	Morphine is the standard used to compare the analgesic effect of other opioids (T)	64 (34.2)	74 (39.6)	49 (26.2)	
3	The extent of the disease determines the method of pain treatment (F)	51 (27.3)	100 (53.5)	36 (19.3)	
4	Adjuvant therapies are important in managing pain (T)	180 (96.3)	3 (1.6)	4 (2.1)	
6	During the last days of life, the drowsiness associated with electrolyte imbalance may decrease the need for sedation (T)	74 (39.6)	61 (32.6)	52 (27.8)	
7	Drug addiction is a major problem when morphine is used on a long‐term basis for the management of pain (F)	26 (13.9)	158 (84.5)	3 (1.6)	
8	Individuals who are taking opioids should also follow a bowel regime (T)	143 (76.5)	13 (7.0)	31 (16.6)	
10	During the terminal stages of an illness, drugs that can cause respiratory depression are appropriate for the treatment of severe dyspnea (T)	62 (33.2)	63 (33.7)	62 (33.2)	
13	The use of placebos is appropriate in the treatment of some types of pain (F)	18 (9.6)	157 (84.0)	12 (6.4)	
14	In high doses, codeine causes more nausea and vomiting than morphine (T)	91 (48.7)	13 (7.0)	83 (44.4)	
15	Suffering and physical pain are synonymous (F)	174 (93.0)	8 (4.3)	5 (2.7)	
16	Demerol is not an effective analgesic in the control of chronic pain (T)	67 (35.8)	59 (31.6)	61 (32.6)	
18	Manifestations of chronic pain are different from those of acute pain (T)	172 (92.0)	5 (2.7)	10 (5.3)	
20	Pain threshold is lowered by anxiety or fatigue (T)	112 (59.9)	45 (24.1)	30 (16.0)	
	Psychosocial and spiritual care				25.67%
5	It is crucial for family members to remain at the bedside until death occurs (F)	3 (1.6)	178 (95.2)	6 (3.2)	
11	Men generally reconcile their grief more quickly than women (F)	91 (48.7)	42 (22.5)	54 (28.9)	
19	The loss of a distant or contentious relationship is easier to resolve than the loss of one that is close or intimate (F)	50 (26.7)	115 (61.5)	22 (11.8)	

Note

T: the answer of the question is “true”; F: the answer of the question is “false.”

### 4.3 Attitudes towards death and caring for dying patients

For students’ attitudes towards death, on average, students showed high scores on neutral acceptance (mean = 3.98; *SD* 0.51) (Table 2). Therefore, most of students in this study regarded death as a natural part of life. For attitudes towards caring for dying patients, students’ overall FATCOD‐C mean score was 101.34 (*SD* 7.75), ranging from 74–125. Most students (*N* = 130, 69.52%) showed a positive attitude towards caring for dying patients according to the cut‐off point 65%. In the FATCOD‐C, nine of 30 items (3, 4, 7, 8, 11, 13–15, 26) had mean scores of less than 3, indicating negative attitudes among students (Table 3). Remarkably, among the above nine items, seven items (3, 8, 11, 13–15, 26) reflected that students were discomfort, especially in the direct care of dying patients and discussion death and emotional reactions related to patients’ impending death. The rest 21 items in the FATCOD‐C had mean scores above 3 ranging from 3.01–4.28, indicating neutral‐to‐moderate positive attitudes among students. The two items that had the highest means in the FATCOD‐C were item 21 “It is beneficial for the dying person to verbalize his or her feelings” and item 18 “Families should be concerned about helping their dying member make the best of his/her remaining life,” and the two items that had the lowest means were item 8 “I would be upset when the dying person I was caring for gave up hope of getting better” and item 26 “I would be uncomfortable if I entered the room of a terminally ill person and found him/her crying.”

**TABLE 2:** Mean scores on the DAP‐R‐C subscales

Scale	Subscales	Mean	*SD*
DAP‐R‐C^a^	Fear of death	2.97	0.61
	Death avoidance	3.07	0.73
	Neutral acceptance	3.98	0.51
	Approach acceptance	2.76	0.56
	Escape acceptance	2.64	0.73

Note

DAP‐R‐C: the Chinese version of the Death Attitude Profile‐Revised.

^a^ The item range for the DAP‐R‐C = 1–5.

**TABLE 3:** The results of the Chinese version of the Frommelt Attitude Toward Care of Dying Scale

Scale item	Mean	*SD*
1. Giving nursing care to the dying person is a worthwhile learning experience	4.20	0.57
2. Death is not the worst thing that can happen to a person	3.85	0.83
3. I would be uncomfortable talking about impending death with the dying person	2.78	0.88
4. Nursing care for the patient's family should continue throughout the period of grief and bereavement	2.90	1.02
5. I would not want to be assigned to care for a dying person	3.01	0.83
6. The nurse should not be the one to talk about death with the dying person	3.13	0.86
7. The length of time required to give nursing care to a dying person would frustrate me	2.79	0.74
8. I would be upset when the dying person I was caring for gave up hope of getting better	2.12	0.65
9. It is difficult to form a close relationship with the family of a dying person	3.06	0.74
10. There are times when death is welcomed by the dying person	3.54	0.80
11. When a patient asks, ‘‘Nurse am I dying?’’, I think it is best to change the subject to something cheerful	2.65	0.88
12. The family should be involved in the physical care of the dying person	4.24	0.65
13. I would hope the person I’m caring for dies when I am not present	2.73	0.91
14. I am afraid to become friends with a dying person	2.93	0.91
15. I would feel like running away when the person actually died	2.87	0.89
16. Families need emotional support to accept the behavior changes of the dying person	4.16	0.62
17. As a patient nears death, the nurse should withdraw from his/her involvement with the patient	3.56	0.78
18. Families should be concerned about helping their dying member make the best of his/her remaining life	4.27	0.68
19. The dying person should not be allowed to make decisions about his/her physical care	3.56	0.85
20. Families should maintain as normal an environment as possible for their dying member	4.25	0.54
21. It is beneficial for the dying person to verbalize his or her feelings	4.28	0.54
22. Nursing care should extend to the family of the dying person	4.04	0.74
23. Nurses should permit dying persons to have flexible visiting schedules	3.98	0.72
24. The dying person and his/her family should be the in‐charge decision makers	3.89	0.79
25. Addiction to pain relieving medication should not be a nursing concern when dealing with a dying person	3.08	1.14
26. I would be uncomfortable if I entered the room of a terminally ill person and found him/her crying	2.28	0.69
27. Dying persons should be given honest answers about their condition	3.02	0.73
28. Educating families about death and dying is not a nursing responsibility	3.66	0.76
29. Family members who stay close to a dying person often interfere with the professionals’ job with the patient	3.06	0.82
30. It is possible for nurses to help patients prepare for death	3.48	0.83

### 4.4 Palliative care self‐efficacy

The mean total self‐efficacy score was 1.96 (*SD* 0.54) ranging from 1–3.42, indicating that students had low level of self‐efficacy in delivering palliative care (Table 4). Seven of 12 items (1, 3, 6, 7, 8, 9 & 10) had a mean score of self‐efficacy <2, ranging from 1.50–1.99, demonstrating that students needed further basic instruction or close supervision/coaching to perform palliative care in those areas. Specifically, out of the seven items, four items were related to symptom management including the management of pain, delirium, nausea/vomiting and breathlessness; one item was informing the available support services; one item was answering the queries about the dying process; and one item was answering patients’ questions about the effects of certain medications.

**TABLE 4:** Mean scores on the Chinese version of the Palliative Care Self‐Efficacy Scale

Scale	Subdimension	Mean	*SD*
Self‐efficacy		1.96	0.54
	Psychosocial support (item: 1–6)	1.98	0.55
	Symptom management (item: 7–12)	1.95	0.69

### 4.5 Correlations among study variables

Examinations of associations among knowledge, death attitudes, attitudes towards caring for the dying and self‐efficacy resulted in some statistically significant correlations (Table 5). However, no significant correlation was found between knowledge with death attitudes, attitudes towards caring for the dying and self‐efficacy. Fear of death was moderately positively correlated with death avoidance (*r* = .648; *p* < .01). Low positive correlations were found between approach acceptance and both death avoidance (*r* = .296; *p* < .01) and escape acceptance (*r* = .482; *p* < .01). There was a low negative correlation between fear of death and attitudes towards caring for the dying (*r* = −.278; *p* < .01).

**TABLE 5:** Correlations among knowledge, attitudes and self‐efficacy regarding palliative care

	Knowledge	Death attitudes					FATCOD‐C
		Fear of death	Death avoidance	Neutral acceptance	Approach acceptance	Escape acceptance	
**Knowledge**							
**Death attitudes**							
Fear of death	−0.101						
Death avoidance	−0.080	**0.648** ^b^					
Neutral acceptance	0.010	**−0.147** ^a^	−0.010				
Approach acceptance	−0.054	**0.255** ^b^	**0.296** ^b^	0.037			
Escape acceptance	0.024	−0.076	−0.078	−0.004	**0.482** ^b^		
**FATCOD‐C**	0.098	**−0.278** ^b^	**−0.239** ^b^	**0.205** ^b^	0.017	0.062	
**Self‐efficacy**	−0.035	−0.014	−0.084	0.081	0.005	**0.170** ^a^	**0.195** ^b^

Note

FATCOD‐C: the Chinese version of the Frommelt Attitude Toward Care of the Dying Scale.

^a^ *p* < .05.

^b^ *p* < .01.

## 5 DISCUSSION

Present study showed that nursing students had favourable attitudes towards death and caring for the dying but had low level of knowledge and self‐efficacy about palliative care. Consistently, Pfister et al. (2013) found that nursing home professionals had low level of knowledge and self‐efficacy about palliative care in Germany. Moreover, Nguyen et al. (2014) revealed that oncology nurses in Vietnam also held favourable attitudes towards caring for the dying but had low level of knowledge and perceived self‐competence regarding palliative care. The above findings highlight the need to increase not only nursing students’ or nurses’ knowledge but also their self‐efficacy in performing palliative care in the future training.

Nursing students had a knowledge deficiency regarding palliative care with the mean score of 9.04 on the PCQN‐C in the present study. Compared with the previous studies conducted in nursing students using the PCQN, this score was slightly lower than the mean score obtained from the third and fourth year nursing students in China (10.41) (Li et al., 2015) but higher than the scores obtained from the second, third, fourth and internship year nursing students in Saudi Arabia (5.23) (Aboshaiqah, 2020) and the second, third and fourth year nursing students in Greece (8.2) (Dimoula et al., 2019). Consistently with the present study, the reason for such low scores in the above studies might be the inadequate palliative care education in nursing programmes (Aboshaiqah, 2020; Dimoula et al., 2019; Li et al., 2015). In addition, the academic parameters (year of study) might also influence the knowledge level of the students (Al Qadire, 2014; Dimoula et al., 2019; Jiang et al., 2019). However, it should be noted that the undergraduate nursing programme in Saudi Arabia is 5 years, while the undergraduate nursing programmes in the present study, Li et al. (2015)’s study and Dimoula et al. (2019)’ study are 4 years.

The present study also found that the category which scored the lowest in the PCQN‐C was the psychosocial and spiritual care, consistently with the studies conducted in nurses in Iran (Iranmanesh et al., 2014) and in Spain (Chover‐Sierra et al., 2017). As in the present study, the reason for the weakness in psychosocial and spiritual care in Iranian nurses and in Spanish nurses could be due to that palliative care education is insufficient and less content is related to the psychosocial and spiritual aspects in the educational programmes (Chover‐Sierra et al., 2017; Iranmanesh et al., 2014). In China, although the nursing curriculum framework has changed from medical focus to nursing process‐oriented model, less attention is paid to the teaching of psychosocial and spiritual care. Moreover, Chinese clinical nurses, who commonly teach nursing students in classroom and in practice, have heavy workloads and they generally focus on patient's physical well‐being and without paying enough attention to the psychosocial and spiritual care of patients. Consequently, limited knowledge regarding psychosocial and spiritual care might be delivered to the nursing students during teaching. Therefore, the present findings suggest the need to strengthen psychosocial and spiritual care teaching in the nursing education. However, the present results are inconsistent with Li et al. (2015)’s study who found that the category which scored the highest percentage of correct answers was psychosocial and spiritual care and the category which scored the lowest was the philosophy of palliative care for the Chinese nursing students. This contradiction could be explained as a consequence of the sample differences. By the end of 2010, 120 colleges in China started international nursing classes, which might provide a path for nursing students to work overseas (Gao et al., 2017). The sample in Li et al. (2015)’s study consisted of 46% of students from international nursing specialty and 54% from general nursing specialty; however, nursing students in the present study were all from general nursing specialty. Nursing students from international nursing specialty had more exposure to novel foreign nursing concepts and were found to have significantly higher scores in all categories of the PCQN than nursing students from general nursing specialty (Li et al., 2015).

In addition, the present study found that item 5 in the PCQN‐C “it is crucial for family members to remain at the bedside until death occurs” got the fewest correct answers. Comparing the previous studies using the PCQN, the studies conducted in nursing students in Jordan (Al Qadire, 2014) and in nurses working in long‐term care homes in Ontario, Canada (Brazil et al., 2012), showed opposite responses; however, studies conducted in Korean nurses (Kim & Hwang, 2014) and Spanish nurses (Chover‐Sierra et al., 2017) and Greek nursing students (Dimoula et al., 2019) did reflect similar responses as the present study. The authors of the original PCQN considered that it is difficult to estimate how long a semi‐conscious patient may live and keeping a vigil can make family members exhausted; therefore, the correct answer is FALSE for item 5. The possible reason for students’ incorrect responses for item 5 in the present study is that due to the family oriented Confucian society in China, people mostly agree that family members should accompany the patient as much as they can especially when the patient is severely ill. Consistently, Kim and Hwang (2014) also found that item 5 is considered correct as a good death consists of family presence at the patient's terminal stage of life in Korean culture. Therefore, culture and the essential role of the family in the care of the dying might also contribute to the samples’ responses for item 5. However, nursing education should help students realize that family members are exhausted physically and psychologically in the process of caring for patient and giving support for family members should also be a priority.

For attitudes towards death, in the present study most of the nursing students perceived death as a natural part of life. Consistently, previous studies found that most of Chinese nursing students (Peng et al., 2018) and Turkish nurses working in inpatient clinics (Cevik & Kav, 2013) and Iranian nurses from oncology ward and general wards (Iranmanesh et al., 2008) all viewed death as a natural part of life. However, this result disagreed with the previous study (Wang et al., 2018) reporting that most of the Chinese nurses perceived death as a passage‐way for a happy afterlife and had low scores on neutral acceptance and death avoidance. The main differences between the participants of the present study and Wang et al. (2018)’s study are that most of the nurses in Wang et al. (2018)’s sample had experience in working with dying patients, and 36.9% nurses took a course on death and dying previously. Considering that there are some factors influencing attitudes towards death such as personal and cognitive frameworks, cultural, philosophical, societal and religious belief systems (Rooda et al., 1999), future study can explore death attitudes between nurses and nursing students.

In the present study, most students had a positive attitude towards caring for the dying with the overall FATCOD‐C mean score of 101.34. Compared with the previous studies investigating nursing students’ attitudes using the FATCOD or the FATCOD Form B (FATCOD‐B), this score is slightly higher than the fourth year nursing students in Palestine (FATCOD‐B = 96.96) (Abu‐EL‐Noor & Abu‐EL‐Noor, 2016) and the third and fourth year nursing students in Turkey (FATCOD = 95.2) (Arslan et al., 2014). However, this score is quite lower than the studies conducted with nursing students in the US (FATCOD‐B = 122.95) (Kirkpatrick et al., 2019) and in Sweden (FATCOD = 132) (Henoch et al., 2017). Education and cultural contexts influence the participants’ attitudes towards caring for dying patients (Iranmanesh et al., 2008; Wang et al., 2018). As Abu‐EL‐Noor and Abu‐EL‐Noor (2016) explained, Turkey and Arab countries including Palestine share some religious and cultural values, which might contribute to that the score of Turkish (Arslan et al., 2014) nursing students was close to the score of the Palestinian nursing students in their study. While the possible reason for students’ high score in attitudes towards caring for the dying in the United States and Sweden might be that these two countries have a long history of research and policy in palliative care. Thus, their nursing students may receive relatively sufficient palliative care education. For example, in Henoch et al. (2017)’s study, the selected six universities in Sweden all provide palliative care education for nursing students, though there are differences between these universities in the curricula for the theoretical elements of palliative care education. In addition, the present study showed that students felt discomfort in the direct care of dying patients and facing the death of patients, consistently with the previous study conducted in Greek nursing students (Dimoula et al., 2019). Since caring for dying patients evokes emotional pressure and stress, future training course may need to consider how to prepare students to psychologically deal with the challenges in the process of patient's dying.

In the present study, no significant relationships were found between knowledge with death attitudes, attitudes towards caring for the dying and self‐efficacy. Inconsistently, Dimoula et al. (2019) conducted a study in undergraduate nursing students in Greece using the PCQN and the FATCOD and reported that there was a low positive correlation between knowledge and attitudes towards caring for the dying. This discrepancy might be related to the differences in palliative care education. In our university, students did not receive mandatory education in palliative care and only few contents related to end‐of‐life care were taught; however, students in Dimoula et al. (2019)’s study received formal palliative care education. In addition, this discrepancy might also be related to the cultural differences that talking about death is taboo under Chinese culture and students might feel more stressful to care for dying patients and tend to be reluctant to care for dying patients. The present study also found that there was a significantly low negative relationship between death attitude (fear of death) and attitudes towards caring for the dying, consistently with the results from Wessel and Rutledge (2005) who conducted the study in southern California nurses. This indicates that students with greater fear of death tend to hold less positive attitudes towards caring for the dying. Therefore, death education might be strengthened to reduce students’ fear of death. For correlation between the DAP‐R‐C subscales, the present study found that fear of death was moderately positively correlated with death avoidance, consistently with the previous evidence (Braun et al., 2010; Cevik & Kav, 2013; Wessel & Rutledge, 2005).

### 5.1 Limitations

Firstly, this study was conducted in nursing students from one university; therefore, the findings might not generalize to all nursing students across China. Secondly, this study assessed students’ self‐efficacy rather than their true competencies or skills in practice. Lastly, since this was a cross‐sectional survey, the associations among these variables could not represent causal relationships.

## 6 CONCLUSION

Nursing students in the present study have favourable attitudes towards death and caring for the dying; however, they have low level of knowledge and self‐efficacy regarding palliative care. This could be due to the inadequate palliative care education in nursing programme. Palliative care education should be integrated into nursing curriculum in China and nursing educators should pay special attention to the teaching of psychosocial and spiritual care and strengthening nursing students’ ability to psychologically deal with the challenges in the process of patient's dying. Currently, nursing educators mainly use lecture and direct demonstration methods (Gao et al., 2017) and clinical experiences in palliative care are seldom to provide in the undergraduate nursing education in China. Thus, nursing educators should not only design the proper teaching content but also need to use various teaching strategies to promote active and experiential learning regarding palliative care. The cultural differences of palliative care found in the present study suggest that palliative care education need to be tailored to the cultural background. Future studies can design palliative care course to improve students’ knowledge and competence in delivering palliative care in China.

## CONFLICT OF INTEREST

None declared.

## AUTHOR CONTRIBUTIONS

Yinghua Zhou: Conception and design, and acquisition of data; drafting the manuscript. Qiao Li: Analysis and interpretation of data; Critical revision for important intellectual content. Wei Zhang: Conception and design, and acquisition of data; Critical revision for important intellectual content. All authors have given final approval of the version to be published. Each author have participated sufficiently in the work to take public responsibility for appropriate portions of the content. They are agreed to be accountable for all aspects of the work in ensuring that questions related to the accuracy or integrity of any part of the work are appropriately investigated and resolved.

## Data Availability

The raw data set used to support the findings of the present study is available from the corresponding author upon request.

## References

[nop2635-bib-0001] Aboshaiqah, A. E. (2020). Predictors of palliative care knowledge among nursing students in Saudi Arabia: A cross‐sectional study. Journal of Nursing Research, 28(1), e60 10.1097/jnr.0000000000000301 30499834

[nop2635-bib-0002] Abu‐El‐Noor, N. I. , & Abu‐El‐Noor, M. K. (2016). Attitude of Palestinian nursing students toward caring for dying patients: A call for change in health education policy. Journal of Holistic Nursing, 34(2), 193–199. 10.1177/0898010115596492 26187999

[nop2635-bib-0003] Al Qadire, M. (2014). Knowledge of palliative care: An online survey. Nurse Education Today, 34(5), 714–718. 10.1016/j.nedt.2013.08.019 24067784

[nop2635-bib-0004] Arslan, D. , Akca, N. K. , Simsek, N. , & Zorba, P. (2014). Student nurses' attitudes toward dying patients in central Anatolia. International Journal of Nursing Knowledge, 25(3), 183–188. 10.1111/2047-3095.12042 24957518

[nop2635-bib-0005] Bandura, A. (1997). Self‐efficacy: The exercise of control. W. H. Freeman.

[nop2635-bib-0006] Braun, M. , Gordon, D. , & Uziely, B. (2010). Associations between oncology nurses' attitudes toward death and caring for dying patients. Oncology Nursing Forum, 37(1), E43–E49. 10.1188/10.ONF.E43-E49 20044331

[nop2635-bib-0007] Brazil, K. , Brink, P. , Kaasalainen, S. , Kelly, M. L. , & McAiney, C. (2012). Knowledge and perceived competence among nurses caring for the dying in long‐term care homes. International Journal of Palliative Nursing, 18(2), 77–83. 10.12968/ijpn.2012.18.2.77 22399045

[nop2635-bib-0008] Cain, C. L. , Surbone, A. , Elk, R. , & Kagawa‐Singer, M. (2018). Culture and palliative care: Preferences, communication, meaning and mutual decision making. Journal of Pain and Symptom Management, 55(5), 1408–1418. 10.1016/j.jpainsymman.2018.01.007 29366913

[nop2635-bib-0009] Cevik, B. , & Kav, S. (2013). Attitudes and experiences of nurses toward death and caring for dying patients in Turkey. Cancer Nursing, 36(6), E58–E65. 10.1097/NCC.0b013e318276924c 23151504

[nop2635-bib-0010] Chover‐Sierra, E. , Martinez‐Sabater, A. , & Lapena‐Monux, Y. (2017). Knowledge in palliative care of nursing professionals at a Spanish hospital. Revista Latino‐Americana De Enfermagem, 25, e2847 10.1590/1518-8345.1610.2847 29069265 PMC5656333

[nop2635-bib-0011] Desbiens, J. F. , Gagnon, J. , & Fillion, L. (2012). Development of a shared theory in palliative care to enhance nursing competence. Journal of Advanced Nursing, 68(9), 2113–2124. 10.1111/j.1365-2648.2011.05917.x 22211701

[nop2635-bib-0012] Dimoula, M. , Kotronoulas, G. , Katsaragakis, S. , Christou, M. , Sgourou, S. , & Patiraki, E. (2019). Undergraduate nursing students' knowledge about palliative care and attitudes towards end‐of‐life care: A three‐cohort, cross‐sectional survey. Nurse Education Today, 74, 7–14. 10.1016/j.nedt.2018.11.025 30554033

[nop2635-bib-0013] Frommelt, K. H. (1991). The effects of death education on nurses' attitudes toward caring for terminally ill persons and their families. The American Journal of Hospice and Palliative Care, 8(5), 37–43. 10.1177/104990919100800509 1742142

[nop2635-bib-0014] Gao, Y. , Zhang, P. P. , Wen, S. F. , & Chen, Y. G. (2017). Challenge, opportunity and development: Influencing factors and tendencies of curriculum innovation on undergraduate nursing education in the mainland of China. Chinese Nursing Research, 4(3), 113–116. 10.1016/j.cnre.2017.07.003

[nop2635-bib-0015] Grubb, C. , & Arthur, A. (2016). Student nurses' experience of and attitudes towards care of the dying: A cross‐sectional study. Palliative Medicine, 30(1), 83–88. 10.1177/0269216315616762 26577928

[nop2635-bib-0016] Henderson, A. , Rowe, J. , Watson, K. , & Hitchen‐Holmes, D. (2016). Graduating nurses' self‐efficacy in palliative care practice: An exploratory study. Nurse Education Today, 39, 141–146. 10.1016/j.nedt.2016.01.005 27006046

[nop2635-bib-0017] Henoch, I. , Melin‐Johansson, C. , Bergh, I. , Strang, S. , Ek, K. , Hammarlund, K. , Lundh Hagelin, C. , Westin, L. , Österlind, J. , & Browall, M. (2017). Undergraduate nursing students' attitudes and preparedness toward caring for dying persons – A longitudinal study. Nurse Education in Practice, 26, 12–20. 10.1016/j.nepr.2017.06.007 28648955

[nop2635-bib-0018] Hsu, C. Y. , O’Connor, M. , & Lee, S. (2009). Understandings of death and dying for people of Chinese origin. Death Studies, 33(2), 153–174. 10.1080/07481180802440431 19143109

[nop2635-bib-0019] Hu, K. , & Feng, D. (2016). Barriers in palliative care in China. Lancet, 387(10025), 1272 10.1016/S0140-6736(16)30017-4 27025427

[nop2635-bib-0020] Iranmanesh, S. , Dargahi, H. , & Abbaszadeh, A. (2008). Attitudes of Iranian nurses toward caring for dying patients. Palliative and Supportive Care, 6(4), 363–369. 10.1017/S1478951508000588 19006591

[nop2635-bib-0021] Iranmanesh, S. , Razban, F. , Tirgari, B. , & Zahra, G. (2014). Nurses' knowledge about palliative care in Southeast Iran. Palliative and Supportive Care, 12(3), 203–210. 10.1017/S1478951512001058 23905678

[nop2635-bib-0022] Jiang, Q. J. , Lu, Y. H. , Ying, Y. P. , & Zhao, H. H. (2019). Attitudes and knowledge of undergraduate nursing students about palliative care: An analysis of influencing factors. Nurse Education Today, 80, 15–21. 10.1016/j.nedt.2019.05.040 31203031

[nop2635-bib-0023] Kassa, H. , Murugan, R. , Zewdu, F. , Hailu, M. , & Woldeyohannes, D. (2014). Assessment of knowledge, attitude and practice and associated factors towards palliative care among nurses working in selected hospitals, Addis Ababa, Ethiopia. BMC Palliative Care, 13(1), 6 10.1186/1472-684X-13-6 24593779 PMC3975866

[nop2635-bib-0024] Kim, S. , & Hwang, W. J. (2014). Palliative care for those with heart failure: Nurses' knowledge, attitude and preparedness to practice. European Journal of Cardiovascular Nursing, 13(2), 124–133. 10.1177/1474515113519521 24399844

[nop2635-bib-0025] Kirkpatrick, A. J. , Cantrell, M. A. , & Smeltzer, S. C. (2019). Relationships among nursing student palliative care knowledge, experience, self‐awareness and performance: An end‐of‐life simulation study. Nurse Education Today, 73, 23–30. 10.1016/j.nedt.2018.11.003 30472406

[nop2635-bib-0026] Li, L. , Hong, F. F. , & Liu, W. J. (2015). The investigation on the cognition in palliative care of nursing undergraduates. Chinese General Practice Nursing, 13(28), 2776–2778. (in Chinese).

[nop2635-bib-0027] Liu, W. J. , Wang, Y. , Yan, C. M. , & Wang, R. X. (2015). The influence of hospice care education on undergraduate nursing students' attitude towards death and end‐of‐life care. Chinese Journal of Nursing Education, 12(2), 103–106. (in Chinese).

[nop2635-bib-0028] Munro, B. H. (2001). Statistical methods for health care research (4th ed.). Lippincott.

[nop2635-bib-0029] Mutto, E. M. , Errázquin, A. , Rabhansl, M. M. , & Villar, M. J. (2010). Nursing education: The experience, attitudes and impact of caring for dying patients by undergraduate Argentinian nursing students. Journal of Palliative Medicine, 13(12), 1445–1450. 10.1089/jpm.2010.0301 21155639

[nop2635-bib-0030] Nguyen, L. T. , Yates, P. , & Osborne, Y. (2014). Palliative care knowledge, attitudes and perceived self‐competence of nurses working in Vietnam. International Journal of Palliative Nursing, 20(9), 448–456. 10.12968/ijpn.2014.20.9.448 25250550

[nop2635-bib-0031] Park, M. , Yeom, H. A. , & Yong, S. J. (2019). Hospice care education needs of nursing homestaff in South Korea: A cross‐sectional study. BMC Palliative Care, 18(1), 20 10.1186/s12904-019-0405-x 30755208 PMC6373091

[nop2635-bib-0032] Peng, Y. , Zhao, L. , Shen, M. D. , Ling, L. , & Zou, H. J. (2018). Analysis of death attitude of undergraduate nursing students and its influencing factors. Chinese Nursing Research, 32(3), 380–383. (in Chinese)

[nop2635-bib-0033] Pfister, D. , Markett, S. , Müller, M. , Müller, S. , Grützner, F. , Rolke, R. , Kern, M. , Schmidt‐Wolf, G. , & Radbruch, L. (2013). German nursing home professionals' knowledge and specific self‐efficacy related to palliative care. Journal of Palliative Medicine, 16(7), 794–798. 10.1089/jpm.2012.0586 23701034

[nop2635-bib-0034] Phillips, J. , Salamonson, Y. , & Davidson, P. M. (2011). An instrument to assess nurses' and care assistants' self‐efficacy to provide a palliative approach to older people in residential aged care: A validation study. International Journal of Nursing Studies, 48(9), 1096–1100. 10.1016/j.ijnurstu.2011.02.015 21377679

[nop2635-bib-0035] Rooda, L. A. , Clements, R. , & Jordan, M. L. (1999). Nurses' attitudes toward death and caring for dying patients. Oncology Nursing Forum, 26(10), 1683–1687. 10573685

[nop2635-bib-0036] Ross, M. M. , McDonald, B. , & McGuinness, J. (1996). The palliative care quiz for nursing (PCQN): The development of an instrument to measure nurses' knowledge of palliative care. Journal of Advanced Nursing, 23(1), 126–137. 10.1111/j.1365-2648.1996.tb03106.x 8708208

[nop2635-bib-0037] Sun, Y. Q. , Sun, B. F. , Li, X. M. , Zhao, J. , & Zhang, P. (2011). A qualitative study on the reasons for cancer families’ choosing whether to inform patient the truth. Medicine and Philosophy, 32(6), 77–78. (in Chinese).

[nop2635-bib-0038] Tang, L. , Li, Y. X. , Zhou, L. J. , Meng, X. L. , & Zhao, J. J. (2015). Effect of death education curriculum on nurses' attitudes towards caring for the dying. Shanghai Nursing, 15(4), 12–15. (in Chinese).

[nop2635-bib-0039] Tang, L. , Zhang, L. , Li, Y. X. , Zhou, L. J. , Cui, J. , Meng, X. L. , & Zhao, J. J. (2014). Validation and reliability of a Chinese version Death Attitude Profile‐Revised (DAP‐R) for nurses. Journal of Nursing Science, 29(14), 64–66. (in Chinese).

[nop2635-bib-0040] Wang, C. C. , Whitehead, L. , & Bayes, S. (2016). Nursing education in China: Meeting the global demand for quality healthcare. International Journal of Nursing Sciences, 3(1), 131–136. 10.1016/j.ijnss.2016.02.009

[nop2635-bib-0041] Wang, L. , Li, C. , Zhang, Q. , & Li, Y. (2018). Clinical nurses' attitudes towards death and caring for dying patients in China. International Journal of Palliative Nursing, 24(1), 33–39. 10.12968/ijpn.2018.24.1.33 29368558

[nop2635-bib-0042] Wessel, E. M. , & Rutledge, D. N. (2005). Home care and hospice nurses’ attitudes toward death and caring for the dying: Effects of palliative care education. Journal of Hospice and Palliative Nursing, 7(4), 212–218. 10.1097/00129191-200507000-00012

[nop2635-bib-0043] Wong, P. , Reker, G. , & Gesser, G. (1994). Death Attitude Profile‐Revised: A multidimensional measure of attitudes toward death In Neimeyer R. A. (Ed.), Death anxiety handbook: Research, instrumentation and application (pp. 121–148). Taylor and Francis.

[nop2635-bib-0044] World Health Organization (WHO) (2006). WHO definition of palliative care. Retrieved from https://www.who.int/cancer/palliative/definition/en/

[nop2635-bib-0045] Yin, Z. , Li, J. , Ma, K. E. , Ning, X. , Chen, H. , Fu, H. , Zhang, H. , Wang, C. , Bruera, E. , & Hui, D. (2017). Development of palliative care in China: A tale of three cities. The Oncologist, 22(11), 1362–1367. 10.1634/theoncologist.2017-0128 28739870 PMC5679827

[nop2635-bib-0046] You, L. M. , Ke, Y. Y. , Zheng, J. , & Wan, L. H. (2015). The development and issues of nursing education in China: A national data analysis. Nurse Education Today, 35, 310–314. 10.1016/j.nedt.2014.10.004 25456256

[nop2635-bib-0047] Zheng, R. S. , Guo, Q. H. , Dong, F. Q. , & Owens, R. G. (2015). Chinese oncology nurses’ experience on caring for dying patients who are on their final days: A qualitative study. International Journal of Nursing Studies, 52(1), 288–296. 10.1016/j.ijnurstu.2014.09.009 25445033

[nop2635-bib-0048] Zhou, Y. , & Zhang, W. (2015). Undergraduate nursing students’ knowledge, attitudes and self‐efficacy regarding pain management. Chinese Journal of Nursing, 50(2), 213–217. (in Chinese).

[nop2635-bib-0049] Zou, M. , Xu, Y. , & Yuan, C. R. (2006). Investigation on status quo of grasping knowledge about palliative nursing of nurses in third level and grade A hospitals. Chinese Nursing Research, 20(12A), 3133–3134. (in Chinese).

